# Predator personality and prey behavioural predictability jointly determine foraging performance

**DOI:** 10.1038/srep40734

**Published:** 2017-01-17

**Authors:** Chia-chen Chang, Huey Yee Teo, Y. Norma-Rashid, Daiqin Li

**Affiliations:** 1 Department of Biological Sciences, National University of Singapore, 14 Science Drive 4, 117543, Singapore; 2 Institute of Biological Sciences, Faculty of Science, University of Malaya, 50603 Kuala Lumpur, Malaysia

## Abstract

Predator-prey interactions play important roles in ecological communities. Personality, consistent inter-individual differences in behaviour, of predators, prey or both are known to influence inter-specific interactions. An individual may also behave differently under the same situation and the level of such variability may differ between individuals. Such intra-individual variability (IIV) or predictability may be a trait on which selection can also act. A few studies have revealed the joint effect of personality types of both predators and prey on predator foraging performance. However, how personality type and IIV of both predators and prey jointly influence predator foraging performance remains untested empirically. Here, we addressed this using a specialized spider-eating jumping spider, *Portia labiata* (Salticidae), as the predator, and a jumping spider, *Cosmophasis umbratica*, as the prey. We examined personality types and IIVs of both *P. labiata* and *C. umbratica* and used their inter- and intra-individual behavioural variation as predictors of foraging performance (i.e., number of attempts to capture prey). Personality type and predictability had a joint effect on predator foraging performance. Aggressive predators performed better in capturing unpredictable (high IIV) prey than predictable (low IIV) prey, while docile predators demonstrated better performance when encountering predictable prey. This study highlights the importance of the joint effect of both predator and prey personality types and IIVs on predator-prey interactions.

Predator-prey interactions are an important component in ecological communities. Whilst predators develop certain behavioural traits to increase foraging success, prey also evolve anti-predation traits to increase their survivorship, such as improved detection and improved ability to avoid detection by prey/predators^1^,^2^. These traits are highly diverse in a population, but how the trait variation is maintained remains a prominent question in ecology and evolutionary biology. The predator-prey interactions are dynamic and bidirectional, however, little attention has been given to understanding how behavioural traits of both predators and prey simultaneously determine their interactions and their fitness consequences in the ecological communities.

Individuals show consistent differences in behaviour across time and/or across contexts^3^. For example, some individuals are consistently more aggressive, bolder, more explorative, and/or more active than others. Such personality differences are evident in numerous animal taxa, ranging from invertebrates to vertebrates^4^,^5^. The personality types of individuals are known to influence their fitness, thus selection can act on them. Personalities of either predators or prey are also known to influence predator-prey interactions^3^,^6^,^7^,^8^,^9^. For example, as predators, bolder brown trouts (*Salmo trutta*) have better foraging success than shy ones^10^, and more aggressive spiders of various species tend to attack prey faster compared to less aggressive individuals^11^,^12^,^13^,^14^. As prey, bolder rainbow trouts (*Oncorhynchus mykiss*) have lower survivorship^6^. Although numerous studies have shown that personalities affect fitness, it is still unclear how personality types are maintained over evolution. One explanation is that different personality types may be maintained through life history trade-offs^15^,^16^. For example, bold individuals may be more likely to find food and have higher growth rate than shy individuals, but due to increased exposure to predators, they may also undergo higher mortality^17^. Another hypothesis suggests that the fitness of a given personality type is context dependent^18^. That is, a certain personality type may be favoured in a particular situation but not in another. For example, bold mud crabs (*Panopeus herbstii*) are preferred by predatory blue crabs (*Callinectes sapidus*) while shy mud crabs are preferred by predatory toadfish (*Opsanus tau*)^18^. In a complex community structure with multiple types of predators, prey with different personality types can therefore co-exist in the population.

Apart from personality (i.e., inter-individual behavioural variation), an individual may behave differently in the same situation and the level of such variability is known to differ between individuals^19^,^20^,^21^. For example, in Ward’s damselfish (*Pomacentrus wardi*), intra-individual variability in latency to leave shelter varies across individuals^22^. Such within-individual temporal variability in behaviour has been defined as intra-individual variability (IIV) or predictability^22^,^23^. Whilst an individual’s personality type represents the *average* behaviour of an individual, such as being more aggressive, IIV is to show the *variation* in behaviour of the same individual, such as being more variable^19^. Bell *et al*.^24^ estimated that about 63% of behavioural variation results from IIV, and theoretical studies have suggested that it may be a trait on which selection can also act^19^,^25^, but empirical studies on the effects of IIV on animal fitness are scarce. Intra-individual variability may be a form of adaptive variation in behaviour related to predator-prey interactions either to facilitate trial-and-error learning or to reduce vulnerability to predators^21^. For example, unpredictable (i.e., high IIV) prey have higher survivorship because predators are less likely to learn their predator-avoidance patterns^22^,^26^,^27^. Unpredictable behaviour is predicted to be most efficient at reducing vulnerability to stalking predators that will observe and memorize predictable patterns of a prey’s movement^28^,^29^,^30^. However, it is still unclear how individuals with different IIV levels may have been maintained within a population.

The predator-prey interactions are bi-directional, so the fitness of prey and predators should be dependent on their counterpart’s personality types. Indeed a handful of studies have revealed the joint effects of personality types of both predators and prey on predator foraging performance and prey survivorship^31^,^32^,^33^. The “locomotor crossover hypothesis” predicts that active/bold predators tend to capture inactive/shy prey, whilst inactive/shy predators tend to capture active/bold prey^34^. This has been supported by empirical studies on a system consisting of black turban snails (*Chlorostoma funebralis*) and their predator sea stars (*Pisaster ochraceus*) as well as in another system composed of crickets (*Acheta domesticus*) and their predator jumping spiders (*Phidippus clarus*)^31^,^32^. However, inconsistent with the locomotor crossover hypothesis, bolder crickets (*Gryllus integer*) have higher mortality when encountering bolder black widow spiders (*Latrodectus hesperus*)^33^. Furthermore, how the personality types and IIV may jointly influence predator-prey interactions still remains largely unexplored. Personality types are known to be related to the cognitive styles, such as sampling style, impulsivity, and learning^35^. For example, personality types are related to how likely an animal is to spend time observing the environment, with less observation time by bolder and more aggressive individuals^35^,^36^. When prey encounter more docile predators that spend time on observing prey movement during foraging, being more unpredictable may be a better strategy to defend against predators compared to more predictable prey. In the case of more aggressive predators that do not spend time on inspecting prey behaviour, the high IIV of prey may no longer be advantageous over those with low IIV. Therefore, predator personality types may influence the fitness of prey with different IIV levels, yet this hypothesis has not been tested.

The prior studies that have considered the joint effects of behavioural variation of both predators and prey on foraging performance have only examined the personality effects on their interactions, but no study has simultaneously considered the influence of personality types and IIVs on predator-prey interactions. In this study we addressed this issue using *Portia labiata,* a specialized spider-eating jumping spider (Araneae: Salticidae), as the predator, and *Cosmophasis umbratica,* an ornate jumping spider, as the prey^37^. *Portia labiata* is well known for its high cognitive ability and complicated foraging strategies^38^,^39^,^40^. All the species of *Portia* that have been studied so far are well-known to use trial-and-error tactics to develop a suitable hunting strategy using feedback signals from the prey^41^. The individual difference in terms of the trial-and-error effectiveness may determine not only how likely they could capture prey, but also the predator mortality as a result of the payoff of making errors. Therefore, *P. labiata* is an excellent model to address the research question, which requires predators to observe and respond to the prey’s anti-predation patterns.

In this study, we addressed the question of how personality types and IIVs of both predators and prey might jointly influence predator foraging performance in terms of number of attempts required to succeed in capturing prey. We first measured aggressiveness and the IIV for the predator species *P. labiata* as well as boldness and the IIV for the prey species *C. umbratica* as inter- and intra-individual behavioural variation for further analysis. After the establishment of personality types and IIVs, we then investigated whether the interaction of personality types and IIVs of both predators and prey could predict predator foraging performance. We predicted that when we take both personality types and IIVs of both predators and prey into consideration, both personality types and IIVs of both predators and prey would jointly predict predator foraging performance. Specifically, we predicted that more docile *P. labiata* that are more likely to observe the prey’s anti-predation patterns would have better foraging performance when encountering more predictable (low IIV) *C. umbratica,* On the other hand, more aggressive *P. labiata* would show similar foraging performance for both predictable and unpredictable *C. umbratica* as more aggressive *P. labiata* may not spend time to observe the anti-predation patterns of *C. umbratica*.

## Result

**Figure 1:**
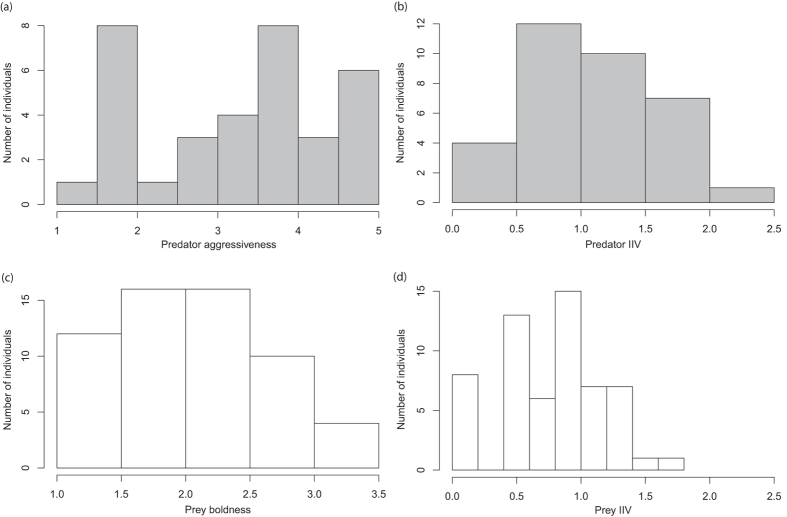
Distribution of individual predator *Portia labiata (n* = 34) aggressiveness score (**a**) and IIV (intra-individual variability) (**b**), and prey *Cosmophasis umbratica (n* = 58) boldness score (**c**) and IIV (**d**) across five trials.

*Portia labiata* showed consistent inter-individual differences in aggressiveness (ICC = 0.32, 95% CI = [0.11, 0.52], *p* < 0.001, *n* = 34), and *C. umbratica* demonstrated consistent inter-individual differences in boldness (ICC = 0.26, 95% CI = [0.11, 0.41], *p* < 0.001, *n* = 58). Individuals exhibited high variation in both average behaviour score as personality (Fig. 1a,c) and intra-individual score as IIV (Fig. 1b,d). Neither body mass nor carapace width was correlated with average personality and IIV (Supplementary Tables S1 and S2). *Portia labiata* aggressiveness was not correlated with the IIV (Supplementary Table S1), whilst *C. umbratica* boldness was positively correlated with the IIV (Supplementary Table S2).

**Figure 2:**
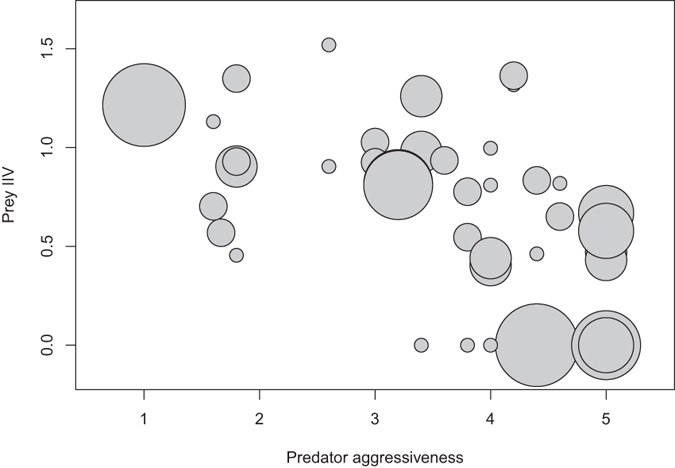
An interaction plot showing the joint effects of predator *Portia labiata* aggressiveness and prey *Cosmophasis umbratica* IIV (intra-individual variability) on the number of attempts to succeed (*n* = 39). The size of the circle represents the number of attempts where the bigger circles indicate more attempts made by a predator to succeed in capturing a prey. The range of number of attempts to succeed is from one to six.

In 39 out of 62 (62.9%) predation trials, predators successfully capture prey within an hour. For those that successfully captured prey, *P. labiata* spent an average of 19.57± 2.60 (range: 2.13–59.50) minutes and made an average of 2.39 ± 0.25 (range: 1–6) attempts to capture prey. For behavioural variation, when we were only concerned about either personality types or IIVs in the model, none of behavioural variations from either party or the interaction between them could predict foraging performance (Supplementary Tables S3 and S4). If we were concerned about both personality types and IIVs of both parties, only the interaction between predator aggressiveness and prey IIV, but neither predator aggressiveness nor prey IIV alone, predicted foraging performance (Table 1, Fig. 2). When a more docile predator encountered a more predictable prey, the predator made fewer attempts to succeed (Fig. 2), whereas when a more aggressive predator encountered a more unpredictable prey, the predator made fewer attempts to succeed (Fig. 2). The number of trials and the body size ratio between prey and predators did not significantly influence the predator’s foraging performance in all three types of models (Table 1, Supplementary Tables S3 and S4).

## Discussion

In this study, we found that personality types and intra-individual variabilities (IIVs) of both predators and prey jointly influenced *P. labiata*’s foraging performance (i.e., number of attempts to capture prey) during predator-prey interactions. Specifically, more aggressive predators required fewer attempts to capture more unpredictable (i.e., higher IIV) prey, while more docile predators required fewer attempts to capture more predictable (i.e., lower IIV) prey. However, the foraging performance was not predicted by the personality types or IIVs of either predator or prey alone. This is the first study to show that predator personality type interacts with prey behavioural predictability to influence the predator’s foraging performance.

The way a predator stalks its prey could also be linked to the benefit of being an unpredictable prey. Bednekoff and Lima^29^,^30^ suggested that unpredictable prey can reduce their vulnerability to stalking predators, like our study predator *P. labiata,* because their behaviours are too unpredictable for the predators to obtain accurate information such as the prey’s movement patterns. However, the assumption that predators should spend time observing the patterns of anti-predator behaviour must be fulfilled. In this case, personality type plays an important role in determining whether being an unpredictable prey is a good strategy to evade predators. In general, more docile predators are more likely to spend time and energy on observing their prey to acquire enough information^35^, to improve their ability to capture more predictable prey over more unpredictable prey. Hence, this allows more unpredictable prey to increase their chance of escaping from predation. This may explain why, in our study, more docile *P. labiata* captured lower IIV *C. umbratica* more easily than they captured higher IIV *C. umbratica*. In contrast, when predators are more aggressive, more likely to be impulsive, and unlikely to spend much time observing the prey movement patterns^35^,^36^, unpredictability would not benefit the prey. Nevertheless, further study is needed to test these hypotheses.

In addition, our data also indicate that more aggressive predators were more likely to capture more unpredictable prey than predictable prey. This result is not congruent with our prediction that more aggressive *P. labiata* would show similar foraging performance when they encounter either predictable or unpredictable *C. umbratica*. One possibility is that the IIV may reflect the cognitive ability of evaluating environments^22^. For example, the IIV of hermit crabs is constrained by their cognitive ability because individuals that are unable to evaluate risk properly are more likely to behave inconsistently^19^,^22^. Therefore, in our system, in the presence of more aggressive *P. labiata*, higher IIV *C. umbratica* individuals with lower cognitive ability may not assess and perform their anti-predation strategy efficiently, resulting in more aggressive *P. labiata* capturing them more easily than lower IIV individuals with higher cognitive ability. This suggests that the context-dependent trade-off may be an important mechanism to maintain differences between personality types and predictability within the population.

Previous studies have suggested that in nature personality types could be maintained in the population via context-dependent mechanisms^31^,^32^,^33^. Our study clearly demonstrates that context-dependent mechanisms in predator-prey interactions may also play an important role in maintaining the natural variation of IIV when high or low IIV is advantageous in one context but not the other. The different compositions of predator personality may influence prey behavioural phenotype distribution in a population^18^,^42^. In a population with a high density of more aggressive predators, being a more predictable prey may lead to higher fitness. In contrast, when the population has a high density of more docile predators, being more unpredictable may lead to higher fitness. In nature, multiple predators with different hunting strategies may coexist, suggesting the possibility of maintaining the differences in prey IIV levels. Moreover, our findings can serve as a basis for understanding the potential evolutionary trajectory of IIV via frequency-dependent selection. When the population consists of a higher proportion of more aggressive predators than docile predators, more predictable prey will be selected because more aggressive predators are better at capturing more unpredictable prey. Therefore, the frequency of more predictable prey will increase, subsequently followed by an increase in more docile predators as more docile predators who capture predictable prey better than more aggressive predators do. As a result, the increase in more docile predators will cause the decline in the frequency of more predictable prey. Hence more unpredictable prey will increase in the population, which causes selection favouring more aggressive predators than more docile predators. Such frequency-dependent selection could make the two personality types of predators and predictability of prey co-exist in the population. Future work should focus on the theoretical and empirical study of the evolution of IIV.

In conclusion, this study emphasizes the significance of behavioural predictability and how it can interact with personality type to influence predator-prey interactions. Specifically, this study highlights that: (1) the behavioural predictability of prey plays an important role in predator-prey interactions; and (2) the behavioural variation (i.e., personality type and IIV) of both predators and prey jointly influences foraging performance. Our findings suggest that the interactions between predator and prey behavioural variation could eliminate single trait optima, so no personality type or predictability is always favoured. The joint effect of predator personality type and prey predictability may serve as an important mechanism for the maintenance of behavioural variation in a population. The study of behavioural IIV is still in its infancy and we strongly suggest that more work should be carried out to understand how predictability of individual behaviour can affect ecological communities.

## Material and Methods

### Study subjects and maintenance

Female adult *Portia labiata (n* = 34) and *Cosmophasis umbratica (n* = 58) were collected in the Cameron Highlands (N 4.4641, E 101.3869) in Malaysia, and Ulu Pandan Park Connector (N 1.3126, E 103.7797) in Singapore, respectively, both in 2015. Spiders were kept individually in plastic cylindrical cages (diameter: 6.5 cm; height: 8.5 cm) and housed in a controlled laboratory (80–90% relative humidity; 12:12 h light: dark cycle; lights on at 0800; at approximately 25 ± 1 °C). All spiders were fed 6–7 laboratory-cultured fruit flies (*Drosophila melanogaster*) twice a week and had *ad libitum* access to water. All spiders were photographed using a digital SLR camera (NIKON D800) with a micro lens (MICRO NIKKOR 105 mm 1:2.8 G ED) and flash (SIGMA EM-140DG). The photographs were then analysed with ImageJ 1.49 to measure the carapace width of the spiders to the nearest 0.01 mm. The spider body mass was measured using an electronic balance to the nearest 0.01 mg.

### Aggressiveness assay of the predator *Portia labiata*

To quantify the aggressiveness of *P. labiata*, the reaction to its own mirror image was used. When encountering conspecifics or mirror images, jumping spiders often engage in complex escalating contests^43^. The mirror images were used to minimize confounding factors associated with the difference in a conspecific’s size or mass^44^,^45^. This method was also used to quantify the aggressiveness in the other salticid species *Eris militaris*^46^. Before the test, we placed the housing container (diameter: 6.5 cm; height: 8.5 cm), with the cap removed, in front of a mirror (14 cm × 6.5 cm). Each individual was allowed a maximum of 20 minutes to interact with its own mirror image. The distance between the spider with agonistic displays and the mirror when it stopped approaching was recorded. As the maximal distance was 5 cm, the distance between the spider and mirror was subtracted from 5, so that individuals with higher value are more aggressive. To ensure that their reactions were repeatable over time, this assay was repeated five times with a one-day interval between trials for each individual.

### Boldness assay of the prey *Cosmophasis umbratica*

To quantify the boldness of *C. umbratica*, the reaction of the individual towards an artificial predator model was recorded. A model predator mimic was constructed using blue-tack, with the basic body shape of a spider (cephalothorax and abdomen; length = 1 cm)^46^,^47^. Legs were made from small pieces of paper clips and inserted on each side. The model was attached to a 15 cm long stick. About 10 minutes before the boldness assay, we first destroyed the nest of each prey spider in its housing container so that it would not be in its nest or would not go into its nest when the trial was underway. The boldness assay was carried out in the prey spider housing container instead of a novel environment so that an extra factor of novelty was not added. After 10 minutes of acclimatization, we started the assay by pushing the model towards the spider from 5 cm away at a constant speed of about 1 cm/sec to simulate the approach of a predator. The initial reaction was recorded and scores were given based on the following criteria: (1) run away from the model (least bold), (2) turn and walk away, (3) move backwards or sideways, (4) touch, jump or climb onto the model (most bold). Moving backwards or sideways was treated as a bolder reaction than turning and walking away because spending a longer time visually assessing the novel model is considered bolder behaviour^46^. Each individual was tested five times with one-day intervals in between to check for repeatability.

### Predation trials

To test how personality types and intra-individual variabilities (IIVs) of both predators and prey would jointly affect a predator’s foraging performance, we performed predation trials using one individual predator and one individual prey after the aggressiveness assay of predators and the boldness assay of prey. To standardize the hunger level of predators, all predators were starved for one week before the tests. To standardize the body size ratio between a predator and a prey item, we tried to pair the prey size to about half the predator size. Prior to the trial, we put one individual predator and one individual prey into separate 5-ml syringes. A cork was used to cap the opening of the syringe and the syringes were inserted into the arena, which consisted of a plastic petri-dish (diameter: 9 cm; height: 1.5 cm) with two gaps at the circumference to release the predator and prey through the syringes. The two individuals were given 10 minutes inside the syringe for acclimation before the corks were removed. Full-spectrum glasses, which allowed UV light to pass through, were placed on top of the petri-dish. The trial began upon the release of the predator and the prey through the syringes into the petri-dish. The two gaps were then closed with a white paper flap.

The trial ended when the predator captured the prey or after one hour if the prey survived. We recorded the number of attempts to capture the prey and if the predator successfully captured the prey. The number of attempts required to succeed was used to indicate a predator’s foraging performance as a higher number of attempts indicates lower foraging performance. Between trials, the petri-dishes and syringes were cleaned with 75% ethanol to remove any chemical cues left behind by the spiders. A total of 34 predator *P. labiata* were used and each was tested twice (total trials: 68), but they were never tested with the same prey individual more than once. Due to the small sample size of prey *C. umbratica*, we reused prey if they survived in the previous predation trials (i.e., out of 58 prey *C. umbratica*, 45 were tested only once, eight were tested twice, and one was tested three times). All interactions were recorded with a digital video camera (JVC GZ-MG50AG). All predation trials were conducted in a dark room with full spectrum lights (i.e., 300–700 nm) from fluorescent tubes (Hitachi BL/B, 20 W) suspended 1.5 m above the experimental apparatus. Spectral illumination including the UV range was used because UV vision is present in both prey *C. umbratica* and predator *P. labiata* and also to simulate natural conditions in the wild^48^,^49^,^50^.

### Statistical analysis

Since two predators lost one of their legs before the predation trials, which impeded their movements, four trials involving these two predators and four prey *C. umbratica* were excluded from the data analyses. We also excluded two trials from the data analyses since in these two trials predator size was five times larger than the prey size, which was much larger than the predator/prey size ratio of the rest of trials where predator size was about 1.25–2 times larger than the prey size. Therefore, a total of 62 trials were included in the final dataset, of which, 39 resulted in predators successfully capturing the prey, and thus were used in the data analysis. In 23 trials, predators did not manage to capture their prey within an hour. We considered these trials as a failure and excluded them from the data analysis.

We used a linear mixed effect model (LMM) to quantify the individual variation of predator aggressiveness and prey boldness with log transformation. Carapace width and trial numbers were coded as fixed effects and spider identity was coded as a random effect. The adjusted repeatability was calculated as between-ID variance divided by the sum of between-ID variance and residual variance^51^. To examine the significant individual differences, likelihood ratio tests were used to compare between a full model and one model without a random effect. The personality type was calculated as the mean of the five scores from the repeated trials in the aggressiveness assay of *P. labiata* and the boldness assay of *C. umbratica* for further analysis. The IIV was calculated using the method from Stamps *et al*.^22^. The residuals for each individual for each behavioural trial were obtained from mixed effect models with ID as a random intercept and trial number as a random slope, and then the residual individual standard deviation (riSD) was calculated^22^. A pair-wise Spearman’s rank correlation test was performed to examine the correlation between personality type (mean), IIV (riSD), carapace width and body mass.

Generalized linear mixed effect models (GLMM) with a Poisson error structure and Laplace approximation were used to evaluate the effect of behavioural variations (personality types and IIVs) of both predators and prey, on the number of attempts to succeed^52^, using *lme4* package^53^. Behavioural variations of predators and prey (i.e., predator aggressiveness, predator IIV, prey boldness, and prey IIV), trial number (1, 2) and body size ratio (carapace width of prey/carapace width of predator) were coded as main fixed effects and the two way interactions between the behavioural variation of predators and prey were also introduced in the full models. Predator ID was coded as a random effect as all predators were tested twice (number of trials). The fixed effects were mean centred for GLMMs to converge. Three models with different behavioural variations as fixed effects were run separately. First, we only considered the personality types of predators and prey, and the two-way interaction between predator and prey personality types. Second, we only considered the IIVs of predators and prey, and the two-way interaction between predators and prey IIVs. Third, we considered both personality types and IIVs of predators and prey, and all possible two-way interactions between predators and prey. No over-dispersion was found in any models. Homoscedasticity and normality of residuals were checked. All models were run in R 3.2.4^54^ and the R code was provided in the supplementary materials.

### Ethical note

The experiments comply with the current legal requirements of Singapore in which the research was conducted, and with all National University of Singapore guidelines (OSHM/PI/13/FOS-289). Although no spiders are restricted by animal protection laws in Singapore, we obtained a National Parks Board permit (NP/RP10-055) for collection. We hand collected a minimal number of spiders for the experiments and kept them in the laboratory under conditions similar to their natural environmental, as described above. The research was based on behavioural observations and all of the experiments with spiders were non-invasive.

**Data accessibility**. Data will be deposited in the Dryad repository at the point of publication.

## Additional Information

**How to cite this article**: Chang, C.-c. *et al*. Predator personality and prey behavioural predictability jointly determine foraging performance. *Sci. Rep.* **7**, 40734; doi: 10.1038/srep40734 (2017).

**Publisher's note:** Springer Nature remains neutral with regard to jurisdictional claims in published maps and institutional affiliations.

## Supplementary Material

Supplementary Materials

## Figures and Tables

**Table 1:** Generalized linear mixed effect models showing the effects of fixed effects on the number of attempts to succeed when both personality types and intra-individual variabilities (IIVs) of both predators *Portia labiata* and prey *Cosmophasis umbratica* were included in the models (*n* = 39).

Fixed effects	Estimate	SE	Z	*P*
Intercept	0.734	0.122	6.017	<0.0001
Trial number	0.030	0.118	0.256	0.798
Body size ratio*	−0.063	0.129	−0.491	0.623
Predator aggressiveness	−0.007	0.119	−0.605	0.545
Predator IIV	0.165	0.126	1.314	0.189
Prey boldness	0.142	0.154	0.918	0.359
Prey IIV	−0.134	0.149	−0.903	0.366
Predator aggressiveness × prey boldness	−0.055	0.159	−0.343	0.731
Predator IIV × prey IIV	−0.096	0.172	−0.558	0.577
Predator IIV × prey boldness	0.087	0.107	0.813	0.416
**Predator aggressiveness × prey IIV**	**−0.321**	**0.124**	**−2.580**	**0.009**

^*^Body size ratio = carapace width of prey/carapace width of predator. Bold indicates the significant value. The full model was step-wise simplified by dropping the least significant interaction term.
