# Supplementary material for: Predator personality and prey behavioural predictability jointly determine foraging performance
Source: Sci Rep. 2017 Jan 17;7:40734. doi: 10.1038/srep40734 (PMC5240143; doi:10.1038/srep40734)
Supplement: Supplementary Materials [file srep40734-s1.pdf]

Supplementary Materials:

Predator personality and prey behavioural predictability jointly determine foraging performance

Chia-chen Chang[1], Huey Yee Teo[1], Y. Norma-Rashid[2] and Daiqin Li[1*]

[1] Department of Biological Sciences, National University of Singapore, 14 Science Drive 4, Singapore 117543

[2] Institute of Biological Sciences, Faculty of Science, University of Malaya, 50603 Kuala Lumpur, Malaysia

Table S1. The correlation among behavioural variation (aggressiveness and intra-individual variation, IIV) and body size (carapace width (CW) and mass) in the predator *Portia labiata* (n = 34). The upper part of the table shows the correlation coefficient and the lower part of the table shows the *p*-value.

	Aggressiveness	IIV	Mass	CW
Aggressiveness	-	-0.31	-0.02	0.02
IIV	0.08	-	-0.04	-0.13
Mass	0.90	0.84	-	**0.62**
CW	0.92	0.45	**< 0.0001**	-

Bold indicates the significant correlation

Table S2. The correlation among behavioural variation (boldness and intra-individual variability, IIV) and body size (carapace width (CW) and mass) in the prey *Cosmophasis umbratica* (n = 58). The upper part of the table shows the correlation coefficient and the lower part of the table shows the *p*-value.

	Boldness	IIV	Mass	CW
Boldness	-	**0.41**	0.04	-0.03
IIV	**0.001**	-	-0.15	0.07
Mass	0.77	0.26	-	**0.32**
CW	0.84	0.62	**0.02**	-

Bold indicates the significant correlation

Table S3. Generalized linear mixed effect models showing the effects of fixed effects on the number of attempts to succeed when only predator *Portia labiata* and prey *Cosmophasis umbratica* personalities were included in the models (n = 39).

**Fixed effect**	**Estimate**	**SE**	**Z**	***P***
Intercept	0.814	0.120	6.810	<0.0001
Trial number	-0.052	0.115	-0.456	0.649
Body size ratio*	-0.105	0.117	-0.898	0.369
Predator aggressiveness	-0.078	0.109	-0.715	0.475
Prey boldness	0.047	0.109	0.431	0.666
Predator aggressiveness × prey boldness	-0.217	0.116	-1.875	0.06

* Body size ratio= carapace width of prey/carapace width of predator.

Table S4. Generalized linear mixed effect models showed the effects of fixed effects on the number of attempts to succeed when only the intra-individual variability (IIV) of predators and prey were included in the models (n = 39).

**Fixed effects**	**Estimate**	**SE**	**Z**	***P***
Intercept	0.862	0.112	7.686	<0.0001
Trial number	-0.038	0.109	-0.342	0.732
Body size ratio*	-0.045	0.109	-0.417	0.677
Predator IIV	0.050	0.118	0.425	0.671
Prey IIV	-0.020	0.115	-0.177	0.860
Predator IIV × prey IIV	-0.009	0.116	-0.082	0.935

* Body size ratio= carapace width of prey/carapace width of predator.

R codes

```
library(lme4)

# aggressiveness

aggressive<-read.table(file.choose(),header=T)

nlevels(aggressive$ID)

agg.m1<-lmer(Distance.score~Carapace.Width+Trial+(1|ID),data=aggressive)

agg.m2<-lm(Distance.score~Carapace.Width+Trial,data=aggressive)

anova(agg.m1,agg.m2) #p<0.0001

summary(agg.m1)

0.8524/(0.8524+1.9184)

agg.ci<-confint(agg.m1, level = 0.95,
            method = "boot",
            nsim = 1000, boot.type = "perc", quiet = FALSE,
            oldNames = TRUE)

agg.ci

0.56^2/(0.56^2+1.57^2)

1.28^2/(1.28^2+1.22^2)


#boldness

bold<-read.table(file.choose(),header=T)

bold.m1<-lmer(log(Boldness)~Bodywidth+Trial+(1|ID),data=bold)

bold.m2<-lm(Boldness~Bodywidth+Trial,data=bold)

anova(bold.m1,bold.m2) #p<0.00001

summary(bold.m1)

0.07/(0.07+0.20)
```

```
bold.ci<-confint(bold.m1, level = 0.95,
        method = "boot",
        nsim = 1000, boot.type = "perc", quiet = FALSE,
        oldNames = TRUE)
bold.ci
0.18^2/(0.18^2+0.49^2)
0.34^2/(0.34^2+0.41^2)

## effect of BT and IIV on predation
predation<-read.table(file.choose(),header=T)
predation<-predation[predation$lose.eg<1,]
predation.size<-predation[predation$Carapace.ratio>0.3,]
N.predation<-predation.size[predation.size$Capture<3600,] # success only
N.predation$sCarapace.ratio<-scale(N.predation$Carapace.ratio)
N.predation$sAgg.<-scale(N.predation$Agg.distance)
N.predation$sAgg.IIV<-scale(N.predation$Agg.IIV.distance)
N.predation$sCosmo.Bold<-scale(N.predation$Cosmo.Bold)
N.predation$sCbold.IIV<-scale(N.predation$Cbold.IIV)
N.predation$sTest<-scale(N.predation$Test)
# both personality and IIV of predator and prey
m1glmm1<-
glmer(Number.of.attacks~sTest+sCarapace.ratio+sAgg.*sCosmo.Bold+sAgg.IIV*sCosmo.Bo
ld+
        sAgg.*sCbold.IIV+sAgg.IIV*sCbold.IIV+(1|Portia.ID)
       ,poisson,nAGQ=1,data=N.predation)
```

```
summary(m1glmm1) # use wald z test to check fixed effects for step-wise simplification
m1glmm2<-update(m1glmm1,~.- sCosmo.Bold:sAgg.)
summary(m1glmm2)
m1glmm3<-update(m1glmm2,~.- sAgg.IIV:sCbold.IIV)
summary(m1glmm3)
m1glmm4<-update(m1glmm3,~.- sCosmo.Bold:sAgg.IIV)
summary(m1glmm4)
m1glmm5<-update(m1glmm4,~.-sAgg.:sCbold.IIV)
anova(m1glmm5,m1glmm4)
#personality of predator and prey
m2glmm1<-
glmer(Number.of.attacks~sTest+sCarapace.ratio+sAgg.*sCosmo.Bold+(1|Portia.ID)
        ,poisson,nAGQ=1,data=N.predation)
summary(m2glmm1)
m2glmm2<-update(m2glmm1,~.-sTest)
summary(m2glmm2)
m2glmm3<-update(m2glmm2,~.-sCarapace.ratio)
summary(m2glmm3)
#IIV of predator and prey
m3glmm1<-
glmer(Number.of.attacks~sTest+sCarapace.ratio+sAgg.IIV*sCbold.IIV+(1|Portia.ID)
        ,poisson,nAGQ=1,data=N.predation)
summary(m3glmm1)
m3glmm2<-update(m3glmm1,~.-sAgg.IIV:sCbold.IIV)
summary(m3glmm2)
```

```
# check for overdispersion
overdisp_fun <- function(model) {
  ## number of variance parameters in
  ## an n-by-n variance-covariance matrix
  vpars <- function(m) { nrow(m)*(nrow(m)+1)/2 }
  model.df <- sum(sapply(VarCorr(model),vpars))+ length(fixef(model))
  rdf <- nrow(model.frame(model))-model.df
  rp <- residuals(model,type="pearson")
  Pearson.chisq <- sum(rp^2)
  prat <- Pearson.chisq/rdf
  pval <- pchisq(Pearson.chisq, df=rdf, lower.tail=FALSE)
  c(chisq=Pearson.chisq,ratio=prat,rdf=rdf,p=pval) }
overdisp_fun(m1glmm4) # not overdispersion
overdisp_fun(m2glmm1)# not overdispersion
overdisp_fun(m3glmm1)# not overdispersion
```
